# Supplementary material for: Liquid marble-derived solid-liquid hybrid superparticles for CO2 capture
Source: Nat Commun. 2019 Apr 23;10:1854. doi: 10.1038/s41467-019-09805-7 (PMC6478824; doi:10.1038/s41467-019-09805-7)
Supplement: Supplementary file 1 — Supplementary Information [file 41467_2019_9805_MOESM1_ESM.pdf]

# Supplementary Information

## Liquid Marble-Derived Solid-Liquid Hybrid Superparticles for $CO_2$ Capture

Hengquan Yang *et al.*

## Supplementary Figures

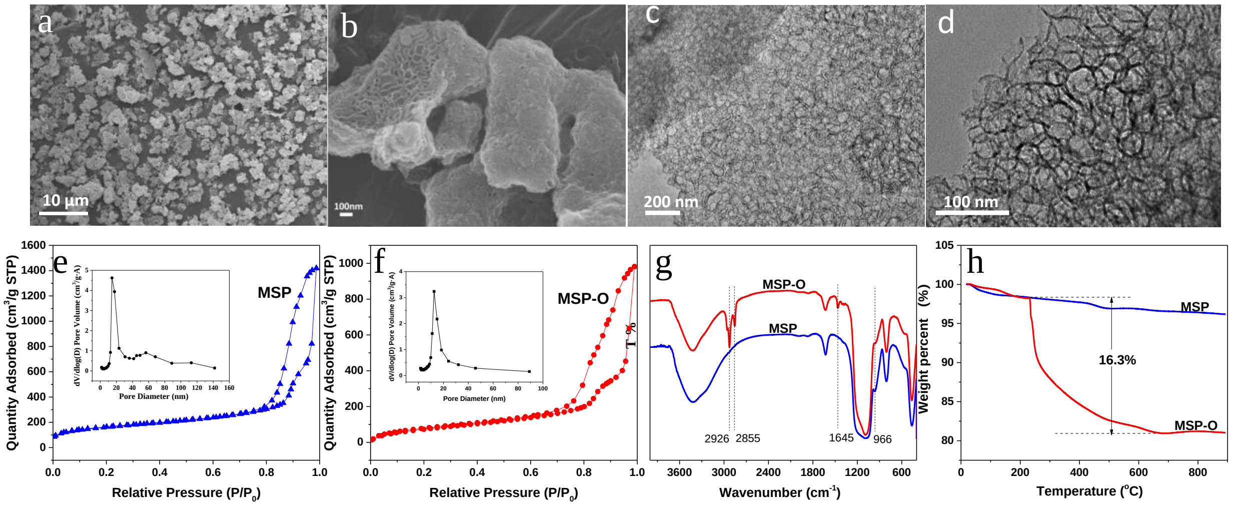


**Supplementary Figure 1 | Characterization of MSP and MSP-O. (a)** SEM image of MSP. (**b**) Magnified SEM image of MSP. (**c**) TEM image of MSP. (**d**) Magnified TEM image of MSP. (**e**) $N_2$ adsorption–desorption isotherm and BJH pore size distribution of MSP (inset). (**f**) $N_2$ adsorption–desorption isotherm and BJH pore size distribution of MSP-O (inset). (**g**) FT-IR spectra of MSP and MSP-O. (**h**) TG curves of MSP and MSP-O. The porosity of MSP is dominantly contributed by the mesoporosity based on the *t*-Plot analysis.

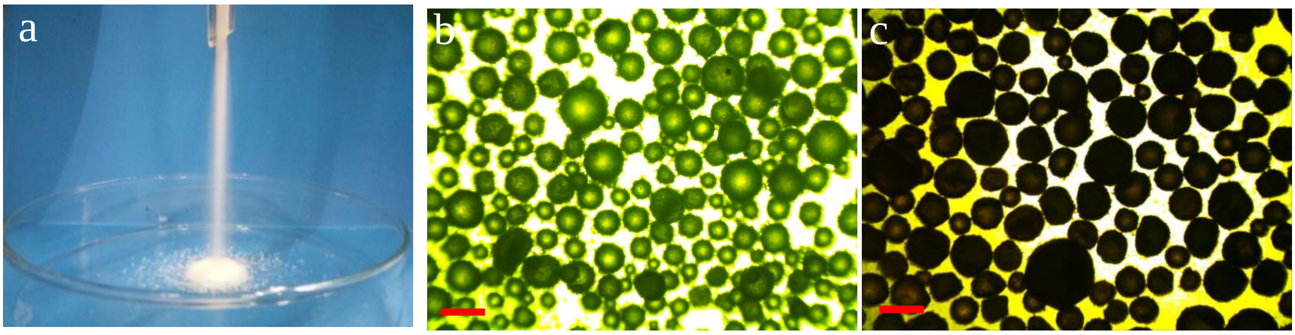


**Supplementary Figure 2 | Characterization of aqueous TEPA marbles. (a)** Appearance of the fresh TEPA marbles. (**b**) Optical microscopy image of the fresh TEPA marbles. (**c**) Optical microscopy image of the TEPA marbles after standing for 15 days. Scale bar = 300 μm.

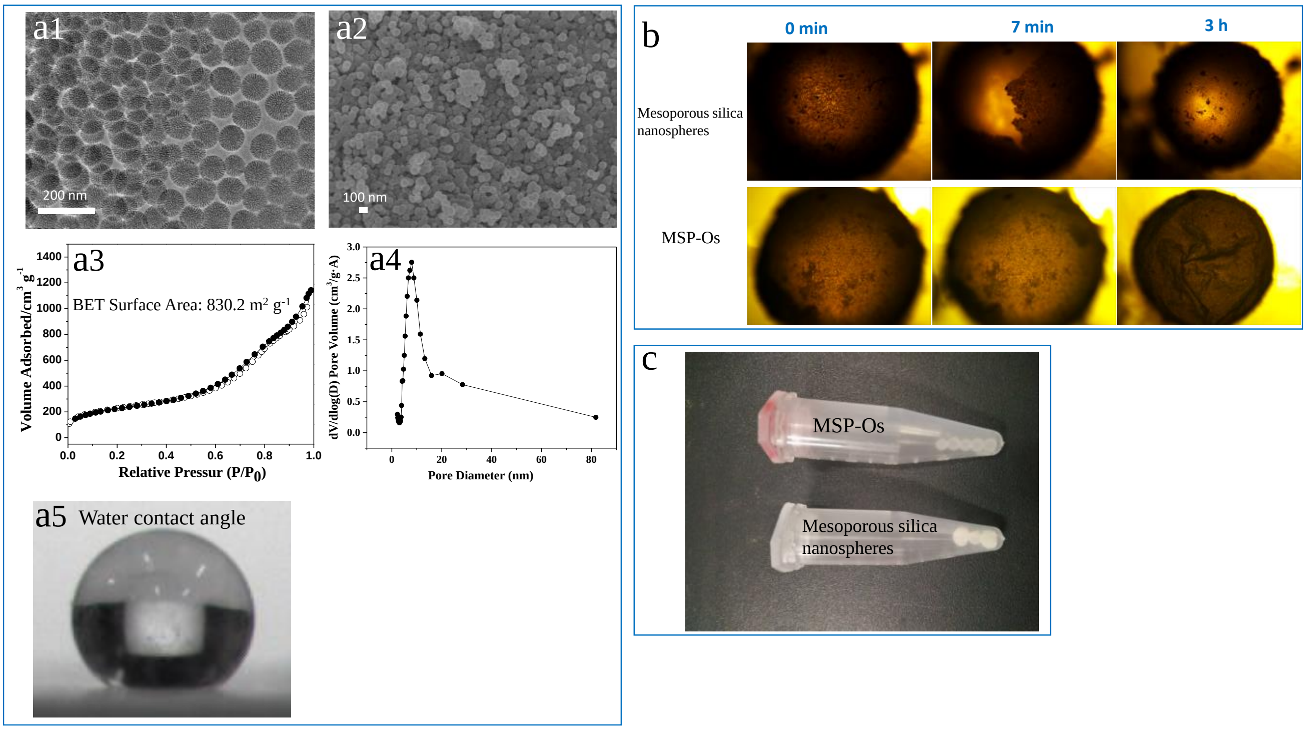


**Supplementary Figure 3 | Distinction between TEPA marbles stabilized by octyl-modified mesoporous silica nanospheres (MSNs) and MSP-Os.** (**a1**) TEM image of MSNs. (**a2**) SEM image of MSNs. (**a3**) $N_2$ adsorption–desorption isotherm of MSNs. (**a4**) BJH pore size distribution of MSNs. (**a5**) Water contact angle of octyl-modified MSNs. (**b**) Optical microscopy images of TEPA marbles stabilized by these two kinds of silicas with different time. (**c**) Appearance of fusion behaviors of two kinds of aqueous TEPA marbles.

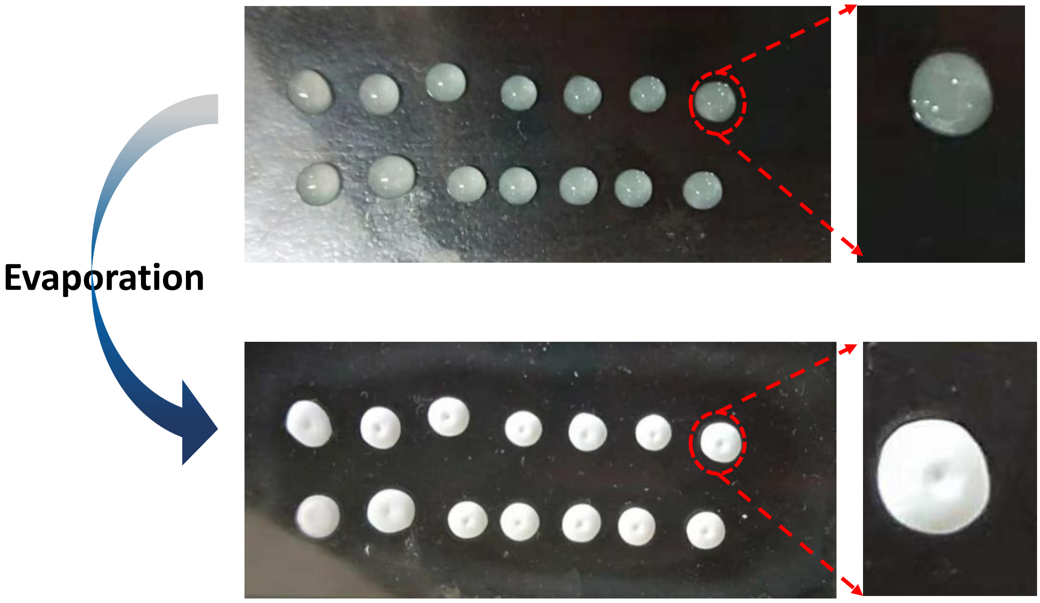


**Supplementary Figure 4 | Appearance of the TEPA/MSP mixture droplets (with 23 wt% MSP) on the glass slide without MSP-Os before and after evaporation.**

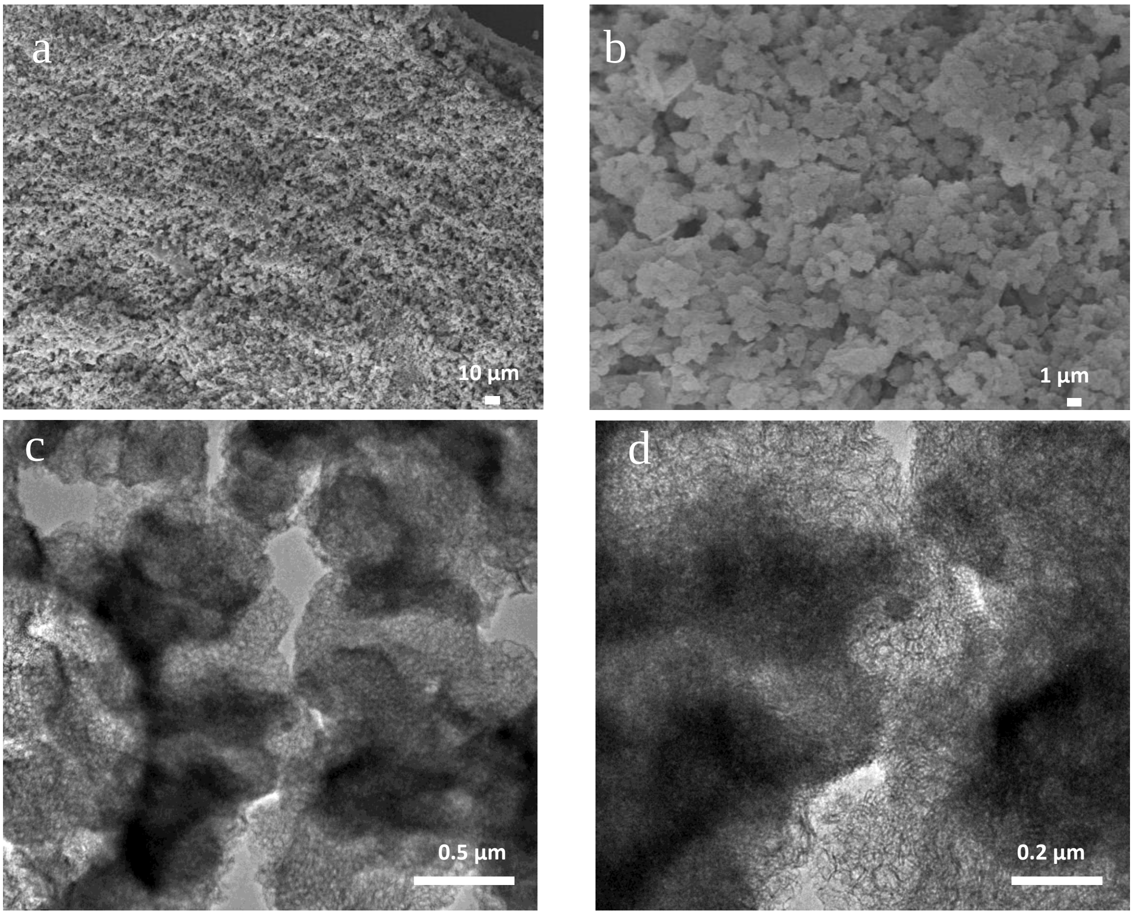


**Supplementary Figure 5 | Characterization of the calcined SLHSP. (a**) SEM image of the cross section of the calcined SLHSP. (**b)** Magnified SEM image of the cross section of the calcined SLHSP. **(c**) TEM image of the calcined SLHSP. (**d**) Magnified TEM image of the calcined SLHSP.

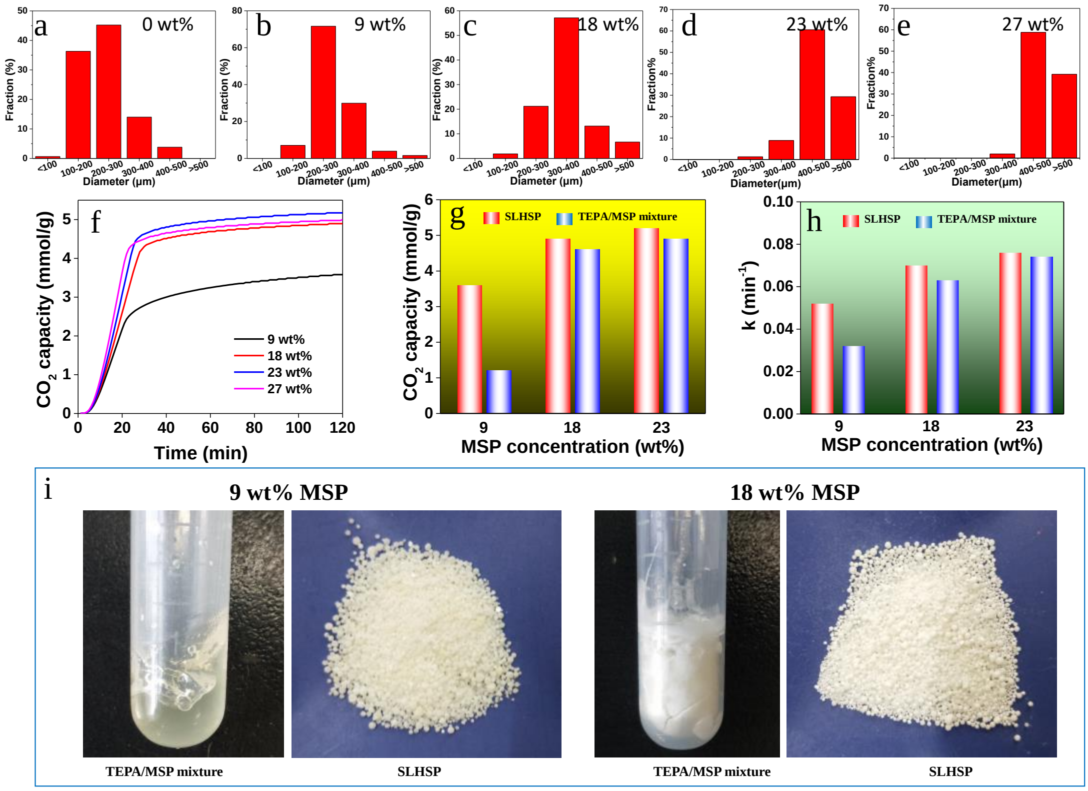


**Supplementary Figure 6 | Size distributions of SLHSPs prepared with different amounts of MSP and comparison of the $CO_2$ sorption capacity and rate between SLHSPs and TEPA/MSP mixture with different amounts of MSP.** (**a**–**e**) Size distributions of the SLHSPs prepared with different amounts of MSP from 0 to 9, 18, 23, and 27 wt%. (**f**) Time-dependent $CO_2$ uptake profiles of SLHSP with different amounts of MSP. (**g**) $CO_2$ sorption capacity of SLHSP and the TEPA/MSP mixture with different amounts of MSP. (**h)** $CO_2$ sorption rate of SLHSP and the TEPA/MSP mixture with different amounts of MSP. (**i**) Appearance of SLHSP and TEPA/MSP with the same amount of MSP (9 and 18 wt%).

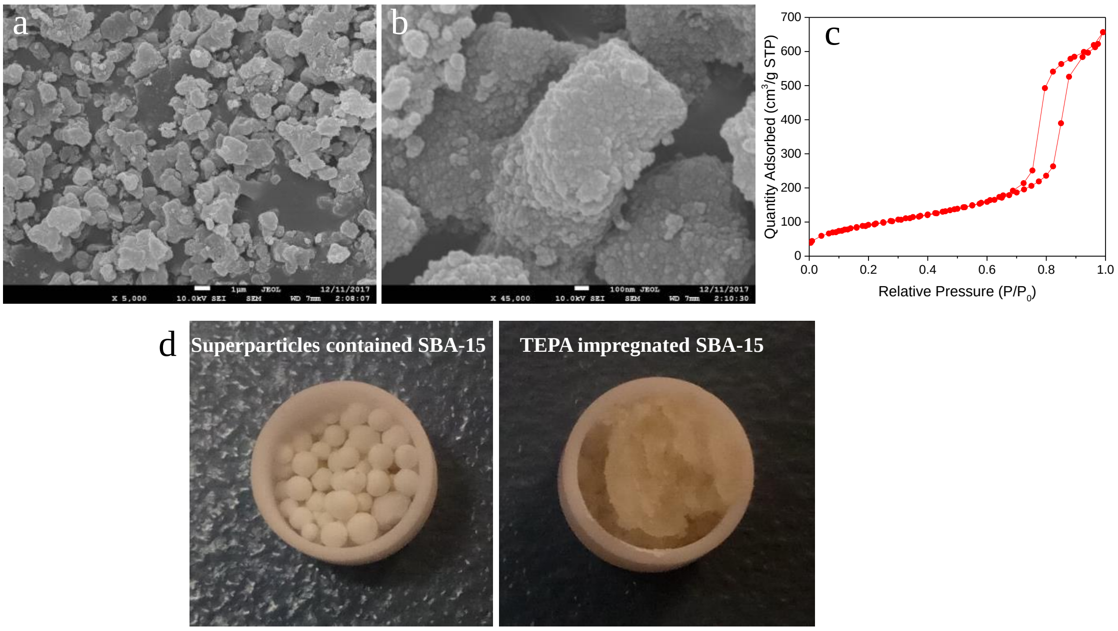


**Supplementary Figure 7 | Characterization of SBA-15 and comparison of appearance between SBA-15 containing SLHSPs and TEPA/SBA-15 mixture. (a)** SEM image of SBA-15. (**b**) TEM image of SBA-15. (**c**) $N_2$ adsorption–desorption isotherm of SBA-15. **(d)** Appearance of SBA-15 containing SLHSP and TEPA/SBA-15 mixture. SBA-15 was synthesized according to the previous literature J. Am. Chem. Soc. 1998, 120, 6024–6036. The porosity of SBA-15 is dominantly contributed by the mesoporosity based on the *t*-Plot analysis.

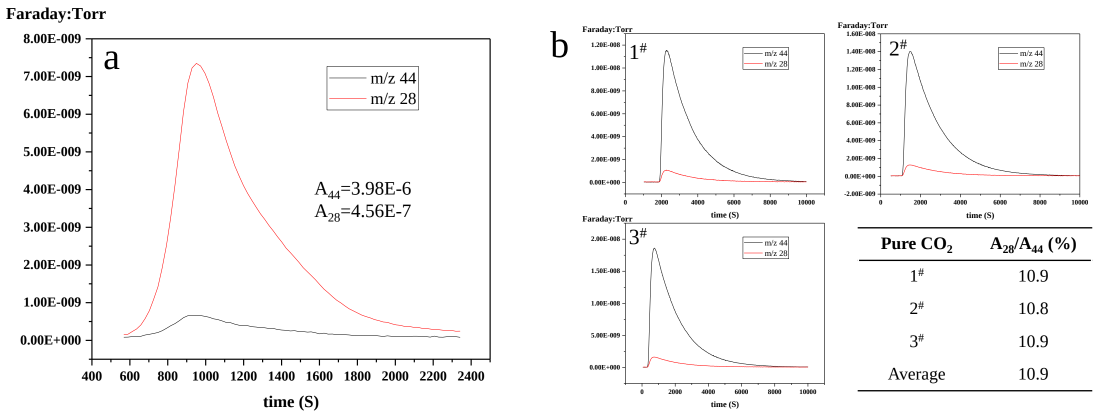


| Pure $CO_2$ | $A_{28}/A_{44}$ (%) |
|---|---|
| 1# | 10.9 |
| 2# | 10.8 |
| 3# | 10.9 |
| Average | 10.9 |

**Supplementary Figure 8 | $CO_2/N_2$ selectivity measurement using TG-MS method. (a)** Mass spectrometry spectra of $CO_2$ and $N_2$ released from the SLHSP that adsorbed $CO_2$. **(b)** Mass spectrometry spectra of m/z 44 and m/z 28 for pure $CO_2$ and the corresponding peak area ratio of m/z 28 to m/z 44. The peak of m/z 44 was assigned to $CO_2$ , and the peak area of m/z 28 was attributed to $N_2$ and fragment of $CO_2$ ($CO^+$, Supplementary Figure 8a)[1]. In order to determine the percentage of $CO_2$ fragment at m/z 28, pure $CO_2$ was also monitored triply by MS. As shown in Supplementary Figure 8b, the average peak area ratio of m/z 28 to m/z 44 is estimated to be 10.9%, which is very close to the value reported in standard spectrum library (11.4%). Thus, the $CO_2/N_2$ selectivity over SLHSP could be calculated according to the equation:

$$\mathrm{Sel}_{CO_2/N_2} = \frac{A_{44}}{A_{28} - A_{44} \times 10.9\%}$$

where $A_{44}$ and $A_{28}$ are the peak areas of m/z 44 and m/z 28, respectively. For example, as shown in Supplementary Figure 8a, $A_{44}= 3.98*10^{-6}$ and $A_{28}=4.56*10^{-7}$, taking these values to the above equation, we can work out the $CO_2/N_2$ selectivity (187).

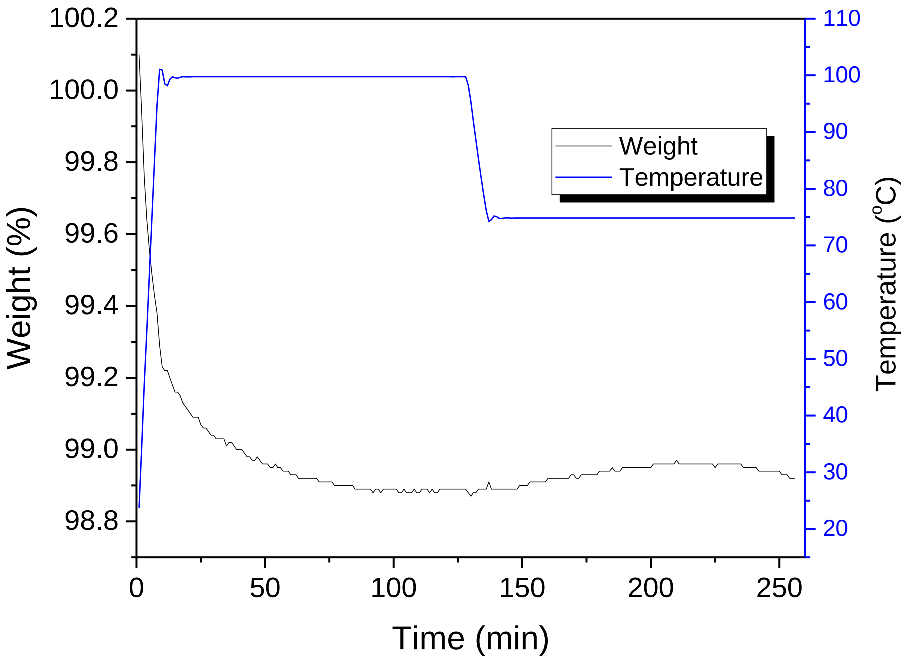


**Supplementary Figure 9 | $CO_2$ sorption of MSP-Os from a simulated flue gas using TGA.**

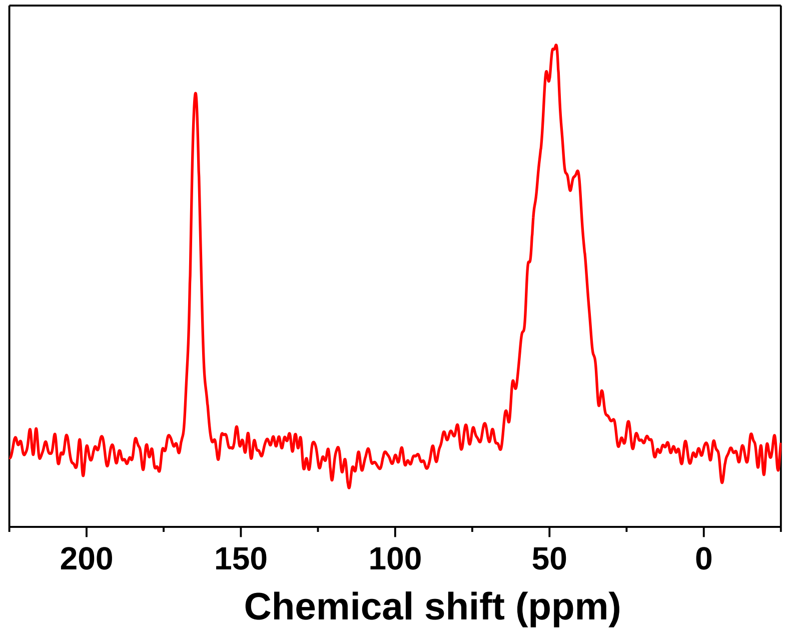


**Supplementary Figure 10 | $^{13}$C CP-MAS NMR spectrum of SLHSP with 23 wt% MSP after $CO_2$ sorption.**

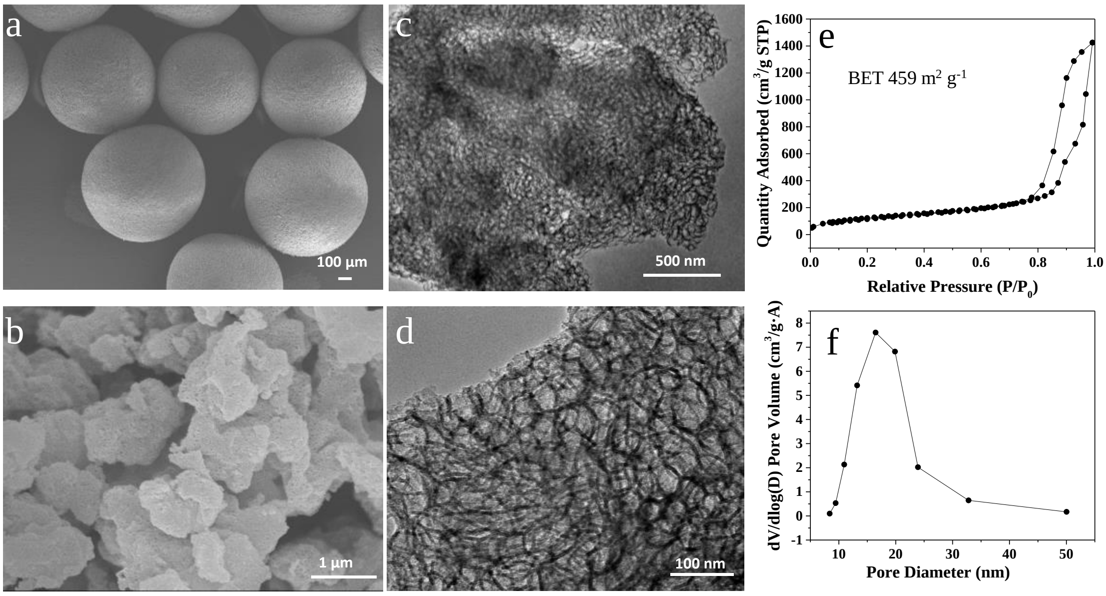


**Supplementary Figure 11 | Characterization of the SLHSPs after 60 cycles (washing with methanol).** (**a**) SEM image. (**b)** Magnified SEM image. **(c**) TEM image. (**d**) Magnified TEM image. (**e**) $N_2$ adsorption–desorption isotherm. (**f**) BJH pore size distribution.

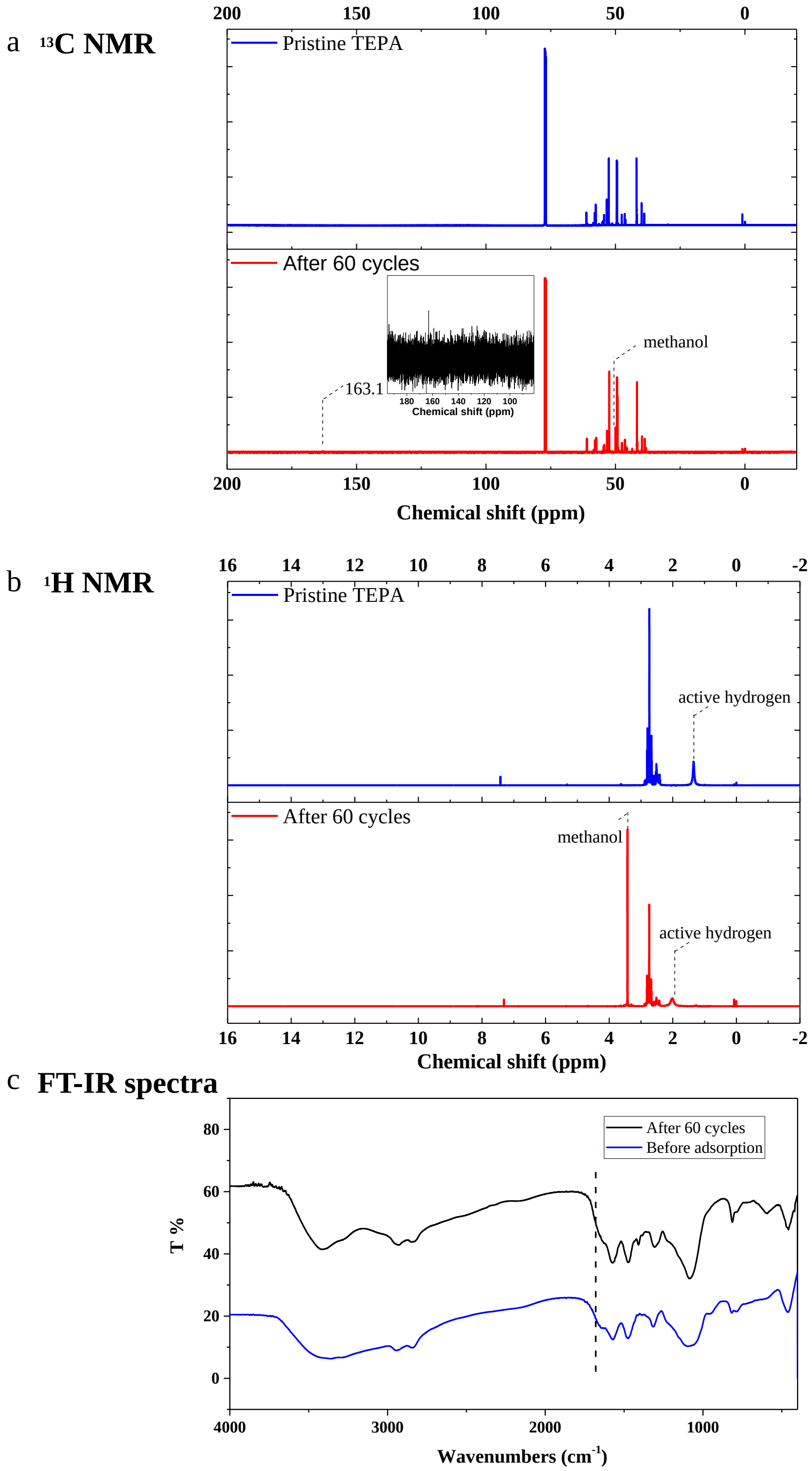


**Supplementary Figure 12 | Characterization of the SLHSPs after 60 cycles. (a)** $^{13}$C NMR spectra of the pristine TEPA and the TEPA collected from the SLHSPs after 60 cycles. **(b)** $^{1}$H NMR spectra of the pristine TEPA and the TEPA collected from the SLHSPs after 60 cycles. **(c)** FT-IR spectra of the fresh SLHSPs and the SLHSPs after 60 cycles.

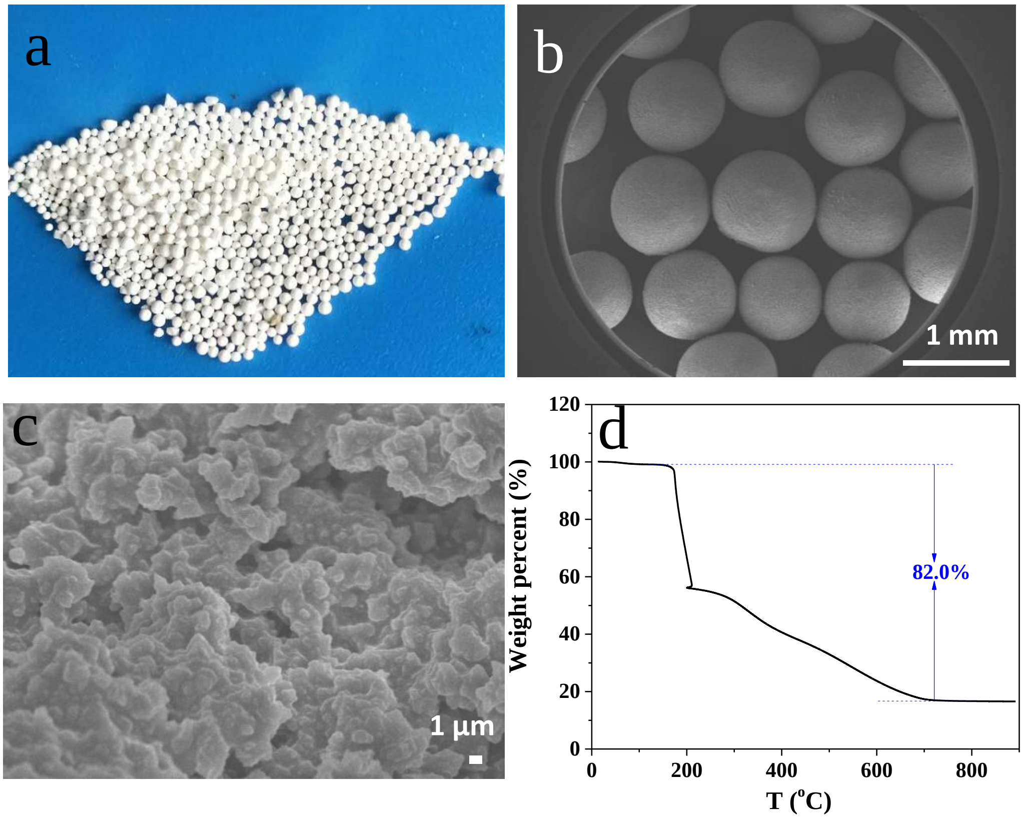


**Supplementary Figure 13 | Characterization of the SLHSPs prepared with PEI.** (**a**) Appearance of SLHSPs prepared with PEI. (**b)** SEM images of PEI-based SLHSPs. (**c**) SEM images of the cross-section of a single PEI-based SLHSP showing the interior structure. (**d**) TGA profile of PEI-based SLHSPs (100 ml $min^{-1}$ air, 10 °C $min^{-1}$).

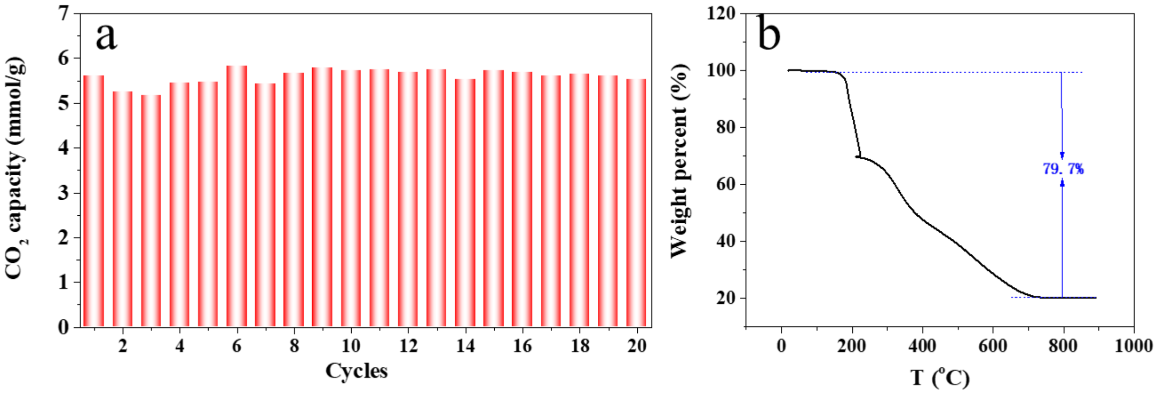


**Supplementary Figure 14 |** (**a**) Multiple cycles of $CO_2$ adsorption–desorption over PEI-based SLHSPs. (**b**) TGA profile of PEI-based SLHSPs after 20 cycles.

## Supplementary Tables

**Supplementary Table 1 |** Three phase contact angles of MSPs modified with different amounts of octyltrimethoxysilane.

| **Octyltrimethoxysilane/MSP (mmol g$^{-1}$)** | **Contact angle (°)** |
|---|---|
| 0 | 20 |
| 0.1 | 88.5 |
| 0.5 | 93 |
| 1 | 113 |
| 2 | 121 |
| 3 | 135 |

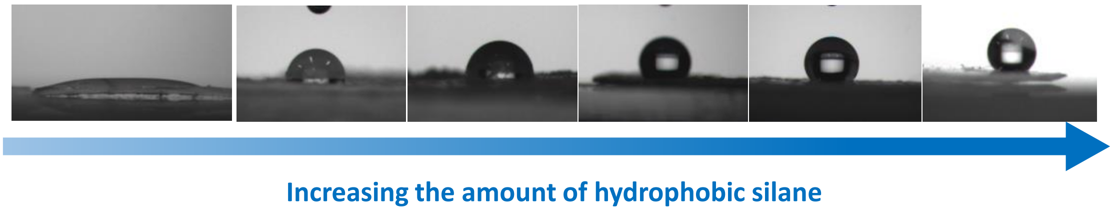


The hydrophobic MSP-Os were prepared via modification with different amounts of octyltrimethoxysilane. The hydrophobility (water contact angle) of MSP-Os was tuned by varying the amount of octyltrimethoxysilane. The measured results show that the water contact angles was increased progressively from 88 $^{o}$ to 135$^{o}$ as the amount of octyltrimethoxysilane increased. MSP-Os with water contact angle of 135$^{o}$ were found to be suitable for preparing the liquid marbles.

**Supplementary Table 2 |** Textural parameters of the pristine MSP and MSP-O.

| Samples | $S^a$ ($m^2$ $g^{-1}$) | $V^b$ ($cm^3$ $g^{-1}$) | Pore size$^c$ (nm) | C element (wt%) |
|---|---|---|---|---|
| Pristine MSP | 539 | 1.7 | 15.5 | – |
| MSP-O | 419 | 1.2 | 12.7 | 8.21 |

$^a$BET surface area. $^b$Single point pore volume calculated at relative pressure of $P/P_0$ = 0.99. $^c$Pore size, BJH method from the desorption branch.

**Supplementary Table 3 |** $CO_2$ sorption capacity and the long-term stability of various sorbents.

| Sorbents | Adsorption capacity (mmol $g^{-1}$) | Cycles | Adsorption method | References |
|---|---|---|---|---|
| 'Dry bases' : 'powdered form' of 50% PEI solution | 4.6 (From the second to tenth cycle: 3.2 mmol $g^{-1}$) | 10 | In a plastic bottle, measured gravimetrically using a balance | *Energy Environ. Sci.*, 2014, **7**, 1786. |
| PEI functionalized with 1,2-epoxybutane, Silica microspheres | 2.2 (15% $CO_2$, 3% $H_2O$, 2% Ar in $N_2$ balance) | 50 | TGA (adsorbent regeneration: pure $CO_2$ at 120 $^{o}$C) | *Nat. Commun.*, 2018, **9**, 726. |
| Immobilization of amino acid ionic liquids into nanoporous microspheres | 1.53 (pure $CO_2$) | 5 | TGA | *J. Mater. Chem. A*, 2013, **1**, 2978. |
| Amine-impregnated millimeter-sized spherical silica foams | 4.3 (pure $CO_2$) | 100 | TGA | *Chem. Eng. J.*, 2015, **259**, 653. |
| TEPA or PEI impregnated mesoporous capsules | 5.5 (10% $CO_2$)<br>7.9 (pre-humidified 10% $CO_2$) | 50 | TGA/packed bed (adsorbents were dispersed in sieved sand) | *Energy Environ. Sci.*, 2011, **4**, 444. |
| PEI 423/10000 impregnated ultra-large silica foam | 5.8 (1 atm pure $CO_2$) | 100 | TGA | *Energy Environ. Sci.*, 2012, **5**, 7368. |
| TEPA or PEI impregnated nanostructured silica | 4.5 (pure $CO_2$) | NO | In glass tube (the weight change) | *Energy Environ. Sci.*, 2010, **3**, 1949. |
| 50% PEI impregnated SBA-15, 'molecular basket' | 3.8 (15 kPa $CO_2$ partial pressure) | 20 | In fixed bed reactor (filled with inert glass beads ) | *J. Am. Chem. Soc.*, 2009, **131**, 5777. |
| PEI impregnated MCM-41 | 4.9 (pure $CO_2$) | 7 | TGA | *Energy Fuels*, 2002, **16**, 1463. |

## Supplementary Methods

### 1. Chemicals

All chemicals were used as received unless otherwise stated. Pluronic P123 copolymer ($EO_{20}PO_{70}EO_{20}$, Mv~5800) were purchased from Sigma-Aldrich. Tetraethyl orthosilicate (TEOS), $(MeO)_3Si(CH_2)_7CH_3$, TEPA and polyethyleneimine (PEI, $M_W$ = 800) were obtained from the Aladdin Company (China). $NH_4F$ was purchased from Tianjin Regant Company (China). Water used in this study was deionized water. All solvents were of analytical quality.

### 2. Material Synthesis

**Synthesis of mesoporous silica particles (MSP).** Mesoporous silica particles were prepared according to the reported method[2]. Typically, 4.8 g $EO_{20}PO_{70}EO_{20}$ (P123) was dissolved in a 168 ml HCl solution (1.30 M). The resultant mixture was stirred at 313 K until the solution became clear, followed by addition of 0.054 g $NH_4F$. After stirring at this temperature for 10 min, a mixture of 21.6418 g octane and 8.0992 g TEOS was then added into the above solution under stirring at 313 K. The resulting mixture was stirred at 313 K for 24 h and then transferred into an autoclave for further condensation at 373 K for 48 h. The resultant solid was collected by filtration, then dried in air and calcined at 823 K for 5 h, eventually leading to MSP.

**Synthesis of hydrophobic MSP-O.** 1.0 g MSPs (dried at 393K for 4 h) were dispersed into 40 ml *p*-xylene. Then 3 mmol trimethylamine and a given amount of $(MeO)_3Si(CH_2)_7CH_3$ (See Supplemantry Table 1) were added into the *p*-xylene suspension. After refluxing at 403 K for 10 h, the resultant material was isolated through centrifugation, then washed five times with toluene and dried under vacuum. After being dried under vacuum, octyl-functionalized MSPs (MSP-Os) were afforded.

**Synthesis of mesoporous silica nanospheres (MSNs).** Mesoporous silica nanospheres were synthesized through a sol-gel process in the presence of CTAC (hexadecyl trimethyl ammonium choloride) as template[3]. 12 g of CTAC was dissolved into a mixture of 112 ml $H_2O$ and 0.36 g triethanolamine at 60 $^oC$. After stirring for 60 min, another mixture of 32 ml cyclohexane and 8 ml TEOS was added into the above solution. After stirring for 12 h. the resultant solid was collected by filtration, and then dried in air and calcined through the same procedure as MSP, eventually yielding MSNs.

**Preparation of FITC-I-labeled MSP-O.** 1.0 g MSP-O and 0.001 mmol $(CH_3CH_2O)_3SiCH_2CH_2CH_2NH_2$ were added into 20 ml toluene. This mixture was stirred under a $N_2$ atmosphere at 60 $^oC$. After 2 h, the resultant solid particles were collected through filtration, washed four times with toluene and dried, resulting in bifunctionalized silica particles. 0.5 g bifunctionalized silica particles and 0.005 g fluorescein isothiocyanate isomer I (FITC-I) were added into 50 ml ethanol. The mixture was stirred overnight at room temperature in the dark. After filtration, the solid was washed five times with ethanol and dried under vacuum, yielding FITC-I-labelled MSP-O.

**Preparation of FITC-I-labeled MSP.** 1.0 g MSP and 0.001 mmol $(CH_3CH_2O)_3SiCH_2CH_2CH_2NH_2$ were added into 20 ml toluene. This mixture was stirred under a $N_2$ atmosphere at 60 $^oC$. After 2 h, the solid particles were collected through filtration, washed four times with toluene and dried, resulting in amino-functionalized silica particles. 0.5 g amino-functionalized silica particles and 0.005 g fluorescein isothiocyanate isomer I (FITC-I) were added into 50 ml ethanol. The mixture was stirred overnight at room temperature in the dark. After filtration, the solid was washed five times with ethanol and dried under vacuum, yielding FITC-I-labelled MSP.

**The procedure for grafting TEPA onto MSP.** 3 mmol (3-bromopropyl)trimethoxysilane was added dropwise to a 10 ml dry toluene solution of TEPA (3.6 mmol) under vigorous stirring. After refluxing for 24 h under a $N_2$ atmosphere, the resultant solution was added to a 50 ml suspension of MSP (1 g). The resultant mixture was further refluxed for another 24 h under a $N_2$ atmosphere. Afterwards, the resultant solid was filtered, washed with toluene and ethanol, and dried at 60 $^oC$ for 12 h, yielding TEPA-grafted MSP.

**3. Preparation of solution TEPA marbles and solid-liquid hybrid superparticles (SLHSP).** Typically, an aqueous solution of 30 wt% TEPA was transferred into a syringe with an orifice diameter of 50 μm and then continuously dropped onto a bed made of MSP-Os with the assistance of the syringe pump followed by rolling of the droplets on the bed, yielding TEPA marbles. To prepare SLHSP, a given amount of MSP was added to 30 wt% TEPA (or PEI) water solution and stirred for about 30 min, forming a suspension. The obtained solid-liquid suspension was transferred into a syringe and continuously dropped onto the bed made of MSP-Os with the assistance of a syringe pump, followed by rolling on the bed, thus compartmentalizing the suspension in individual marbles. Then, slow

evaporation of water inside the marbles caused isotropic shrinkage of the marbles, forming solid-liquid hybrid superparticles (SLHSP). To further prepare SLHSPs with different sizes, the suspension flow rate was changed from 0.05 to 0.15 and then to 0.30 ml $min^{-1}$.

**4. Mechanical stability test.** 0.5 g SLHSPs with 23 wt% MSP were filled in a quartz column (diameter of 0.7 cm and length of 10 cm) and were subjected to be fluidized with $N_2$ at a superficial velocity of 0.3 m $s^{-1}$ for 3 h. Likewise, 0.5 g SLHSPs were packed in a fixed-bed reactor. 2 MPa $N_2$ (25 ml $min^{-1}$) was allowed to continuously pass through this bed for 3 h. After the treatment, the sample was collected for SEM observation.

**5. Nanoindentation.** Agilent G200 nanoindenter with a Berkovich diamond indenter was used for nanoindentation. Prior to actual indentation experiments on organic crystal, the tip stiffness and geometry were determined using Corning 7980 silica reference sample (Nanomechanics S1495-25). The modulus was calculated using the Oliver-Pharr method, where a fit of the unloading curve is used to determine the stiffness, the contact depth, the reduced modulus of the system, and finally the modulus of the sample. The value of the Poisson's ratio was assumed to be 0.3 (typical of anisotropic crystalline solids).

**6. $CO_2$ sorption measurements using TGA method.** $CO_2$ sorption was also performed using a TA Instruments Q600 thermal gravimetric analyzer. 15% $CO_2$ (in $N_2$, V/V) was used as simulated flue gas and pure $N_2$ was used as stripping gas for sorbent regeneration. In a typical sorption process, 10-20 mg sorbent was placed in a platinum sample pan. After being heated to 100 $^oC$ in $N_2$ (25 ml $min^{-1}$) and kept at this temperature for 2 h to remove the adsorbed $CO_2$, the sorbent was cooled down to 75 $^oC$ and equilibrated at this temperature for about 2 h. The gas was then switched to a simulated flue gas at 75 $^oC$ for $CO_2$ sorption. The sorbent capacity (mmol $g^{-1}$) was calculated based on the weight gain of the sorbent.

**7. $CO_2/N_2$ selectivity measurements using thermogravimetric-mass spectrometry (TG-MS).** The $CO_2/N_2$ selectivity was measured with TG-MS analyzer during the course of $CO_2$ desorption. Typically, about 20 mg SLHSP after completing $CO_2$ sorption was placed in a thermal gravimetric analyzer. After sufficiently purging the TG-MS analyzer with argon at room temperature, the sample was heated to 100 $^oC$ for $CO_2$ desorption. The $CO_2$ and $N_2$ released from the SLHSP were monitored with the online

MS analyzer using MID mode (Multiple Ion Detection).

**8. $CO_2$ adsorption–desorption measurements in a fixed-bed reactor.** In a typical adsorption measurement, ~500 mg sorbent was packed in a quartz column (diameter of 0.7 cm and length of 10 cm), at bottom of which was filled with glass wool. Prior to $CO_2$ adsorption, the sorbent was activated at 100 °C for 60 min in dry $N_2$ at a flow rate of 25 ml min$^{-1}$ and then cooled down to 75 °C. Subsequently, the inlet gas wa s switched to 15% $CO_2$ at 25 ml min$^{-1}$. The $CO_2$ concentration of the effluent gas was monitored using an online $CO_2$ analyzer (an A-CMI220 Carbon Dioxide Measurement Instrument) for online determination. After sorption for a certain time, the inlet gas was switched back to $N_2$ and the packed bed was heated to 100 °C for $CO_2$ desorption. Following the same procedure, a blank control measurement was carried out using the bed packed only with glass wool to evaluate the dilution effect from gas changes. The $CO_2$ concentration profile after removal of the background was used for the sorbent capacity calculations.

$$\mathrm{q} = \frac{Q}{M_{ad}} \times \int_0^t (C_o - C_t)dt \times \frac{T_0}{T} \times \frac{1}{V_m}$$

where $q$ is the $CO_2$ sorption capacity (mmol g$^{-1}$), $M_{ad}$ the mass of adsorbents, $t$ is the adsorption time (s), $C_0$ and $C_t$ is is the $CO_2$ concentration of the inlet and outlet, $T$ is the adsorption temperature (K), and $V_m$ is the standard molar volume (22.4 L mol$^{-1}$, $T_0$=0 °C ).

The multiple adsorption–desorption cycles in the presence of moisture were conducted with the same procedure except dilute $CO_2$ were humidified by passing the gases through a water bubbler at room temperature. The relative humidity of the gases was measured using an A-CMI220 relative humidity sensor.

**9. Steam treatment.** The stability of SLHSP under harsh conditions was assessed by performing a steam treatment according to a previously published procedure[4,5]. Typically, a glass tube was filled with 0.4 g of SLHSP and then put into a glass beaker that was filled with approximately 10 ml deionized water. The beaker was then placed in an autoclave. After sealing the autoclave, nitrogen gas was purged through the autoclave for 20 min. The autoclave was then sealed and heated to the desired temperature (106 °C) and kept at the desired temperature for 12 h. The steam pressure inside the autoclave was autogenous and monitored by a pressure transducer. When the autoclave was opened after cooling down to room temperature, no liquid water was observed to have accumulated in the

sample tube, suggesting the solids were contacted with water vapor only and not liquid water. The samples were then transferred to a vacuum oven and dried at 60 °C overnight.

**10. Characterization.** Transmission electron microscopy (TEM) images were obtained on a JEM-2000EX (operated at 200 kV). Scanning electron microscope (SEM) images were obtained using a JSM-7500F electron microscope. The samples were placed on carbon tape and coated with 1 nm platinum for SEM measurements. C and N content analysis was conducted on a Vario EL (Elementar). Solid state NMR spectra were recorded on an Infinityplus 300 MHz spectrometer: for $^{13}C$ CP-MAS NMR experiments, 75.4 MHz resonant frequency, 10.0 kHz spin rate, 5.0 s pulse delay, 1.0 ms contact time, hexamethyl benzene as a reference compound. FT-IR spectra were recorded on a Thermo-Nicolet-Nexus 470 infrared spectrometer. $N_2$ physical sorption was measured with an ASAP2020 volumetric adsorption analyzer. Before measuring, all samples were outgassed at 150 °C under vacuum for 6 h, and the pressure was decreased down to 1 μmHg. The specific surface area was calculated from the adsorption branch in the relative pressure range of 0.04−0.16 using the Brunauer−Emmett−Teller (BET) method. Pore diameters were calculated from the adsorption branch of the isotherm using the BJH method. The total pore volume was measured at a relative pressure of $P/P_0 > 0.99$. Water contact angles (water-solid-air) were measured on KRUSS DSA100. Before measurement, the powder sample was compressed into a pellet (ca. 2 MPa, thickness of approximately 1 mm). A drop of water (1 μL) was injected on the sample pellet. The appearance of the water drop was recorded at ca. 0.1 second, and the value of the contact angle was determined by the photogoniometric method. Aqueous TEPA marbles were mounted on a glass slide and were observed on an optical microscope (XSP-8CA, Shanghai, China). The $CO_2$ capacity of SLHSP and TEPA impregnated materials was conducted on a simulated flue gas using the TA Q600. The $CO_2/N_2$ selectivity of SLHSP was performed on a TG-MS analyzer (TA Q600-HIDEN HPR 20). The adsorption–desorption cycles were monitored in the packed bed was measured, and the outlet was connected the inline $CO_2$ analyzer system (an A-CMI220 Carbon Dioxide Measurement Instrument).

## Supplementary Notes

**Derivation of the criteria for mechanical stability of superparticles.** The stress $\sigma^{(s)}$ in a thin shell of thickness *h*, under the application of an external force per unit area *f*, satisfies the equation

$$h\nabla\sigma^{(s)} + f = 0 \qquad (1)$$

Now consider the above equation, expressed in spherical polar coordinates, for a case where the shell covers a sphere of radius *R* and force *f* is applied along the surface (i.e. in the tangential $\theta$ direction). For such circumstances, the Supplementary Equation 1 becomes

$$\frac{h}{R}\frac{\partial\sigma^{(s)}_{\theta\theta}}{\partial\theta} - f_\theta = 0 \qquad (2)$$

where $f_\theta$ is from now on understood to be the force exerted at the surface of a droplet, by the hydrodynamic flow of the surrounding continuous phase relative to it. For a shell constructed from a material capable of supporting both extensional as well as compressive stresses, the tensile stress in the shell at the top of the droplet will normally be extensional. However, in the case of interest here, we are dealing with a shell comprised of hard spheres. Such spheres, in the absence of any attractive forces between them, can only support compressive stresses. The result of this is that at the upper pole of the droplet ($\theta = 0^0$), where the stress will take its minimum value, the stress is zero and gradually becomes more compressive as one moves along the surface of the droplet towards the lower pole ($\theta = 180^0$). Fixing the value of stress at the upper pole allows one to integrate Supplementary Equation 2 to determine the compressive stresses in the rest of the shell, that is

$$\sigma^{(s)}_{\theta\theta}(\theta) = \frac{R}{h}\int_0^\theta f_\theta \, d\theta \qquad (3)$$

As mentioned above we are specifically interested in $\sigma^{(s)}_{\theta\theta}$ when $f_\theta$ is the result of the flow of the dispersion fluid around the droplet. For this classical Stokes flow problem, the radial and tangential components of the fluid velocity ($v_r$ and $v_\theta$) in the medium outside the sphere are very well established and are given respectively by

$$v_r = -u\cos(\theta)\left[1 - \frac{3R}{2r} + \frac{R^3}{2r^3}\right] \qquad (4a)$$

and

$$v_\theta = u\sin(\theta)\left[1 - \frac{3R}{4r} - \frac{R^3}{4r^3}\right] \qquad (4b)$$

at a distance *r* away from the centre of the droplet in the direction *θ*, respectively. The velocity *u* appearing in the above set of equations represents the velocity flow of the dispersion medium, at points far from our droplet. Now substituting Supplementary Equation 4 in the appropriate equation for the resulting stress tensor in gas, *i.e.*

$$\sigma_{r\theta}^{(f)} = \eta\left[r\frac{\partial}{\partial r}\left(\frac{v_\theta}{r}\right) + \frac{1}{r}\frac{\partial v_r}{\partial \theta}\right] \qquad (5)$$

where *η* denotes the viscosity of the continuous phase, Then the required external applied force, exerted on the surface of the droplet, becomes

$$f_\theta = \sigma_{r\theta}^{(f)} = \frac{3\eta uR}{2}\sin\theta \qquad (6)$$

This in turn, and after substituting in Supplementary Equation 3 and integration, yields

$$\sigma_{\theta\theta}^{(s)}(\theta) = \frac{3\eta u}{2h}(\cos\theta - 1) \qquad (7)$$

At the point of maximum compression (lower pole), the compressive stresses in the shell are thus $3\eta u/h$. This is the point at which the particles making up the shell will first be forced to leave the surface of the droplet. To see this, first consider a particle at this location on the surface at equilibrium. Whether this is a stable or an unstable equilibrium can be ascertained by considering a slight displacement, *Δz*, of the particle away from this position. At equilibrium, contact forces between this particle and its neighbours, though subjecting the particle to compression, all average out and therefore there is no net force. However, as soon as the particle is displaced, a net force of magnitude

$$F_{hyd} = 6\pi\eta u\Delta z \qquad (8)$$

acting in the direction away from the surface will result. For simplicity, we have assumed here that the contact angle of the particles on the interface between the dispersed and the dispersion media is $90^0$, thus making the thickness of the shell *h* ~ size of particles. Opposing this force will be the capillary forces $F_{cap}$ which will be pulling the particle back towards its equilibrium position. For small displacements, the force-displacement relation resulting from such capillary forces is linear, allowing for definition of an effective spring constant. This, often the so called Hooke-de Gennes spring constant $k_s$, was recently calculated by us for particles sitting on the surface of droplets[6,7], and found to be given by

$$k_s = \frac{4\pi\gamma}{2\ln\left(\frac{2R}{h}\right)\pm} \tag{9}$$

where $\gamma$ is the interfacial tension between the dispersed phase and the dispersion medium, and the ± sign in the equation reflects the nature of contact line on the surface of the particles; the subtraction applying to a case where the contact line is pinned at the surface of the particle, while the addition is for when it is free to retreat. Now, it is clear that if the above spring constant is larger than the one found in Supplementary Equation 8, the net result of the two forces is for the particle to be pulled back. In other words equilibrium is stable. This happens when the flow velocity $u^*$ is

$$u^* = \frac{\gamma}{3\eta\left(\ln\left(\frac{2R}{h}\right)\pm\frac{1}{2}\right)} \tag{10}$$

For flow rates above this, any slight displacement of a particle from the surface of the droplet will cause for it to be displaced. With the detachment of particles from their surfaces, the stabilised SLHSP become prone to destabilisation and our superparticles lose their integrity. Therefore, our superparticles can keep stability when the flow rate is less than the limiting flow.

## Supplementary References


1. Ren, X. M., Li, H., Chen, J., Wei, L. J., Modak, A., Yang, H. Q. & Yang, Q. H. N-doped porous carbons with exceptionally high $CO_2$ selectivity for $CO_2$ capture. *Carbon* **114**, 473−481 (2017).

2. Sun, J., Zhang, H., Tian, R., Ma, D., Bao, X., Su, D. S. & Zou, H. Ultrafast enzyme immobilization over large-pore nanoscale mesoporous silica particles. *Chem. Commun.* **12**, 1322−1324 (2006).

3. Shen, D., Yang, J., Li, X., Zhou, L., Zhang, R., Li, W., Chen, L., Wang, R., Zhang, F. & Zhao, D. Biphase stratification approach to three-dimensional dendritic biodegradable mesoporous silica nanospheres. *Nano letters* **14**, 923−932 (2014).

4. Li, W., Bollini, P., Didas, S. A., Choi, S., Drese, J. H. & Jones, C. W. Structural changes of silica mesocellular foam supported amine-functionalized $CO_2$ adsorbents upon exposure to steam. *ACS Appl. Mater. Interfaces* **2**, 3363−3372 (2010).

5. Qi, G., Fu, L. & Giannelis, E. P. Sponges with covalently tethered amines for high-efficiency carbon capture. *Nat. Commun.* **5**, 5796 (2014).

6. Ettelaie, R. & Lishchuk, S. V. Detachment force of particles from fluid droplets. *Soft Matter* **11**, 4251−4265 (2015).

7. Lishchuk, S. V. & Ettelaie, R. Detachment force of particles with pinning of contact line from fluid bubbles/droplets. *Langmuir* **32**, 13040−13045 (2016).
